# Psoriatic Arthritis Without Cutaneous Manifestations: A Case of Diagnostic Delay, Pathologic Progression, and Psychological Burden

**DOI:** 10.7759/cureus.117097

**Published:** 2026-09-28

**Authors:** Paris Tomlinson, Kelsey N Sauers, Emeri Nosal, Brae Davies, Colleen E McGarvey, Johnny Stackhouse

**Affiliations:** 1 Medicine, Edward Via College of Osteopathic Medicine, Auburn, USA; 2 Internal Medicine, Edward Via College of Osteopathic Medicine, Fort Walton Beach, USA; 3 Pediatrics, Edward Via College of Osteopathic Medicine, Huntsville, USA; 4 Obstetrics and Gynecology, Edward Via College of Osteopathic Medicine, Fort Walton Beach, USA; 5 Clinical Affairs, Edward Via College of Osteopathic Medicine, Auburn, USA

**Keywords:** depression, diagnostic delay, differential diagnosis of joint pain, family history, psoriatic arthritis, seronegative spondyloarthritis, without skin lesions

## Abstract

Psoriatic arthritis (PsA) is a chronic inflammatory musculoskeletal disease. Presentations can vary, but common manifestations include peripheral arthritis, dactylitis, enthesitis, nail changes, and skin changes, including psoriasis. Diagnosis is often challenging due to the absence of specific biomarkers, variable clinical presentation, and overlap with conditions such as osteoarthritis and fibromyalgia. These diagnostic obstacles contribute to frequent misdiagnosis and delay in treatment, which, over time, leads to irreversible joint damage, functional impairment, and increased comorbidities.

A 62-year-old woman with a 15-year history of chronic pain attributed to fibromyalgia and osteoarthritis presented to primary care with persistent hand and foot pain and worsening depressive symptoms. Prior treatments failed to provide relief. Imaging and laboratory evaluation were not consistent with her previous diagnoses of fibromyalgia and osteoarthritis. Although she lacked characteristic psoriatic skin lesions, further history revealed a family history of psoriasis in her daughter and grandson. This finding prompted consideration of PsA, and the diagnosis was made based on clinical presentation and history. The patient was started on disease-modifying antirheumatic drug therapy and escitalopram, with significant improvement in both pain and mood within one month.

This case exemplifies the impacts of delayed diagnosis in a patient with PsA without cutaneous manifestations. Common misdiagnoses of PsA can include fibromyalgia, osteoarthritis, and rheumatoid arthritis due to overlapping subjective and objective findings, making diagnosis difficult. This delay leads to prolonged initiation of treatment and thus results in further joint damage, functional decline, and mental health impacts.

Quicker suspicion and thorough history taking in patients with chronic musculoskeletal pain unresponsive to treatment could potentially improve diagnostic delay trends of PsA, even in the absence of cutaneous findings.

## Introduction

Psoriatic arthritis (PsA) is a chronic inflammatory musculoskeletal disease with a prevalence of 1-2 per 1000 individuals [1]. Presentation varies, but common manifestations include peripheral arthritis, dactylitis, enthesitis, nail changes, and psoriasis [2]. Diagnosis is guided by the CASPAR criteria, which require inflammatory articular disease and a score of ≥3 based on features including current or prior psoriasis, family history of psoriasis, psoriatic nail changes, negative rheumatoid factor, dactylitis, or characteristic radiographic findings [3]. CASPAR was developed and validated as a classification tool for use in patients with established inflammatory arthritis, primarily for research and cohort identification, rather than as a stand-alone diagnostic test; in clinical practice, it is applied alongside history, examination, and exclusion of mimicking conditions. Diagnosis is challenging due to the absence of biomarkers, variable presentation, and overlapping symptoms with other conditions, such as osteoarthritis and fibromyalgia [2,4]. These challenges often result in delayed diagnosis, leading to worse patient outcomes.

Diagnostic delay in PsA is well-documented and continues to be a significant clinical concern. Studies report average delays exceeding 5 years [4]. In contrast with other inflammatory arthropathies such as rheumatoid arthritis, PsA is associated with higher rates of misdiagnosis [5]. Osteoarthritis and fibromyalgia are frequent misdiagnoses [4,5]. Untreated disease has been shown to lead to irreversible joint damage and increased psychological burden [4-6]. Even a delay of as little as six months from symptom onset to diagnosis has been associated with greater joint erosion and poorer long-term physical function [4]. 

Chronic pain and functional limitations contribute to depression and reduced quality of life [6]. This relationship underscores the importance of early recognition, particularly in those with persistent symptoms not responding to standard therapy. 

Diagnosis is further complicated by patients lacking cutaneous manifestations, which occurs in approximately 15-20% of cases [7]. In these patients, psoriasis has not yet appeared or may never appear, so clinical suspicion must instead be guided by a thorough history - including family history of psoriasis, a recognized independent risk factor, and CASPAR criteria that can support the diagnosis even in the absence of skin disease - alongside careful evaluation of musculoskeletal symptoms.

This case highlights the diagnostic challenges of PsA in the absence of cutaneous manifestations and illustrates the clinical and psychological burden that may accompany prolonged symptoms and delayed recognition.

## Case presentation

A 62-year-old Hispanic woman presented to her primary care provider with concerns of persistent hand and foot pain and to review imaging studies. She reported a 15-year history of progressively worsening chronic pain despite medical management. Her medical history was significant for fibromyalgia and osteoarthritis, as well as hypertension, hyperlipidemia, and gastroesophageal reflux disease.

The patient described bilateral hand and foot pain, fatigue, and worsening depressive symptoms. Her medications included atorvastatin, chlorthalidone, omeprazole, duloxetine, meloxicam, diclofenac topical gel, and acetaminophen, with minimal relief. Review of systems was notable for chronic musculoskeletal pain and fatigue but negative for rash, joint swelling, fever, nail changes, or gastrointestinal symptoms. She denied any personal history of psoriasis. The available clinical documentation did not characterize the presence or duration of morning stiffness or symptoms of inflammatory back pain. Psychiatric screening using the Patient Health Questionnaire-9 (PHQ-9) [8] revealed severe major depressive disorder with a score of 20, worsened from 14 three years prior. The patient denied suicide ideation or thoughts of harming herself or others.

Physical examination revealed tenderness of the distal interphalangeal (DIP) and proximal interphalangeal (PIP) joints of the hands bilaterally and tenderness at the bilateral Achilles tendon insertions. No dactylitis was observed. No objective joint swelling or synovitis was identified. Dermatologic examination included the scalp, retroauricular regions, umbilicus, intergluteal/natal cleft, and nails of the hands and feet and revealed no cutaneous or nail findings suggestive of psoriasis.

Plain radiographs of the feet demonstrated enthesophyte formation at the Achilles tendon insertion on the calcaneus bilaterally (Figures 1-2) and at the plantar fascia insertion on the left foot (Figure 2). Plain radiography of the right hand demonstrated moderate degenerative changes of the digits, with cortical erosions and periarticular changes at the DIP and PIP joints of the right ring finger, read by radiology as consistent with an inflammatory arthropathy (Figure 3).

**Figure 1:**
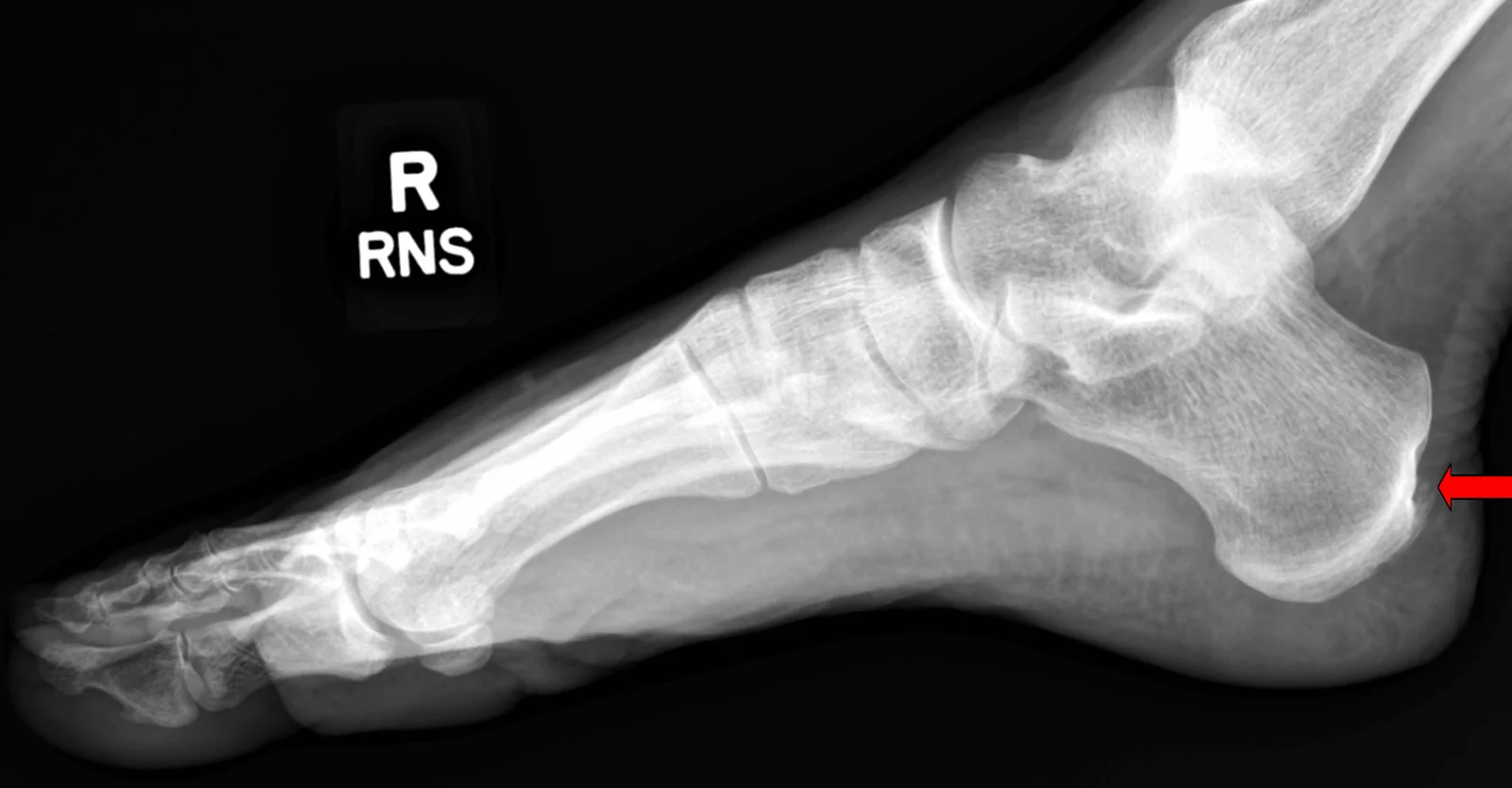
Lateral radiograph of the right foot showing a posterior calcaneal enthesophyte at the Achilles tendon insertion (arrow)

**Figure 2:**
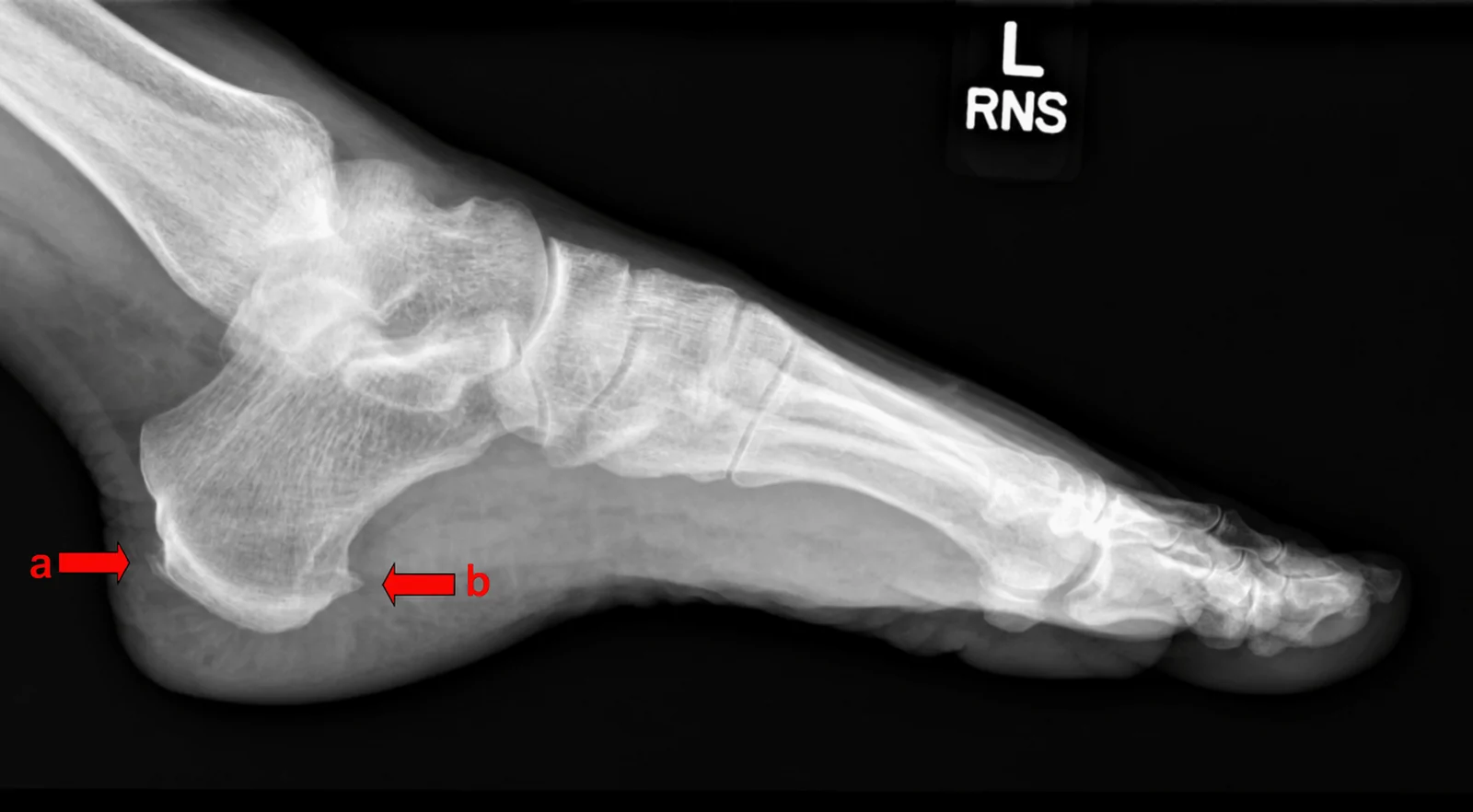
Lateral radiograph of the left foot showing a posterior calcaneal enthesophyte at the Achilles tendon insertion (a) and an inferior calcaneal enthesophyte on the plantar fascia insertion (b)

**Figure 3:**
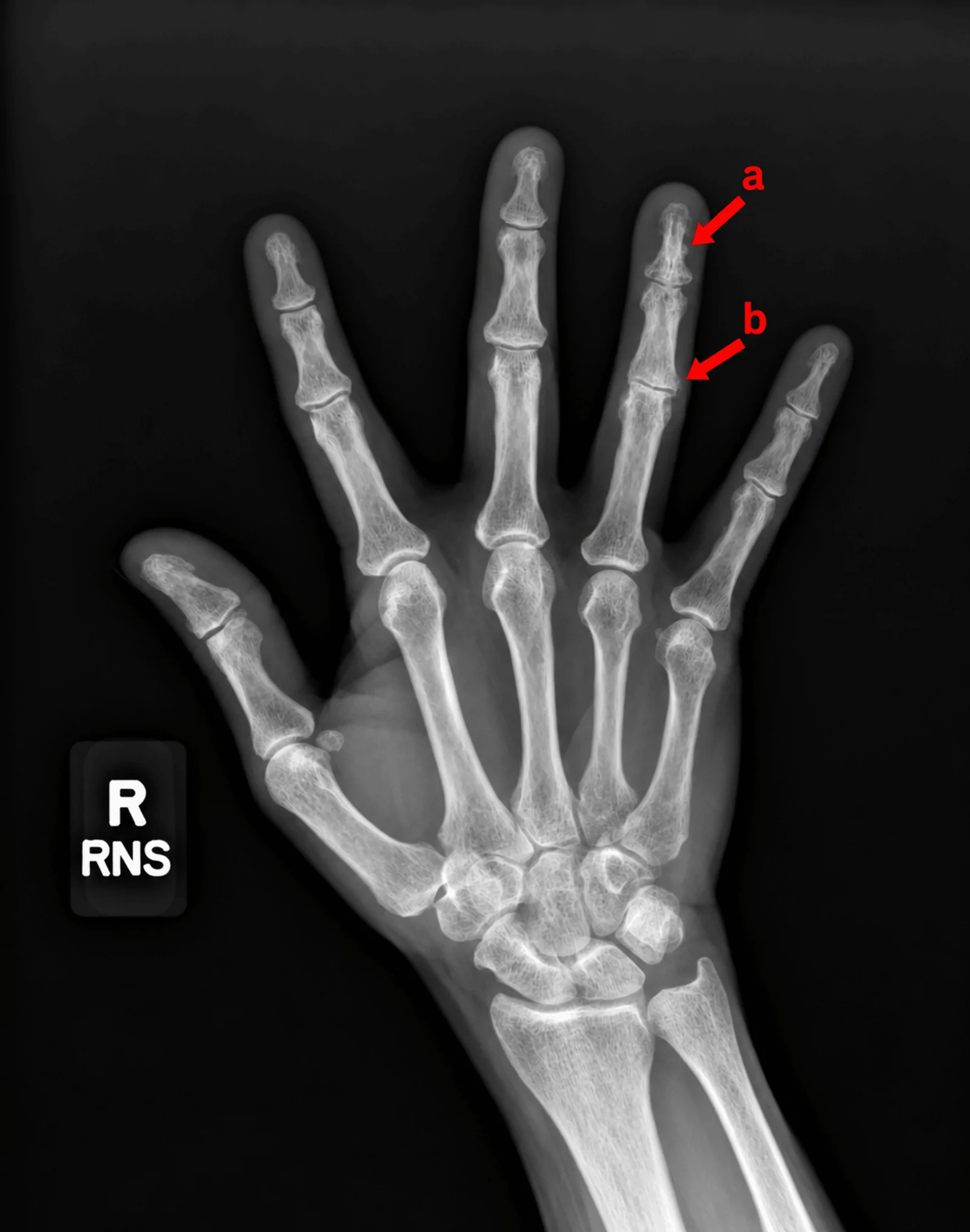
Plain radiograph of the right hand demonstrating periarticular new bone formation at the DIP joint (a) and cortical erosion with joint space narrowing at the PIP joint (b) of the right ring finger, with moderate degenerative changes of the digits DIP: distal interphalangeal; PIP: proximal interphalangeal

Laboratory evaluation demonstrated an erythrocyte sedimentation rate (ESR) of 28 mm/hr, with negative rheumatoid factor and negative anti-cyclic citrullinated peptide (anti-CCP) antibodies.

In the setting of suspected inflammatory articular disease, the patient's family history of psoriasis, negative rheumatoid factor, and periarticular radiographic changes at the right ring finger were considered supportive of PsA and were evaluated using the CASPAR classification framework (Table 1). The CASPAR score was 3, meeting the classification threshold in the setting of inflammatory articular disease. 

**Table 1:** Application of the CASPAR classification criteria in this patient Table created by the authors based on the CASPAR criteria described by Taylor et al. [9]. CASPAR: Classification Criteria for Psoriatic Arthritis *Personal history and family history are scored only if current psoriasis is absent. Juxta-articular new bone formation is defined as ill-defined ossification near joint margins, excluding osteophyte formation, observed on plain radiographs of the hand or foot. Patients are classified as having psoriatic arthritis if they have inflammatory articular disease and accumulate ≥3 points. The CASPAR criteria have a reported sensitivity of approximately 91.4% and specificity of 98.7% [9].

CASPAR feature assessed	Patient finding	Points awarded
Inflammatory articular disease	Present	Required
Current psoriasis	Absent	0
Personal history of psoriasis*	Absent	0
Family history of psoriasis*	Psoriasis in daughter and grandson	1
Psoriatic nail dystrophy	Absent	0
Negative rheumatoid factor	Rheumatoid factor negative	1
Current or previous dactylitis	Absent	0
Juxta-articular new bone formation	Present on radiographic imaging	1
Total CASPAR score	Meets classification threshold	3

Duloxetine was discontinued, and escitalopram was initiated for the management of major depressive disorder. The patient was referred to rheumatology for further evaluation of suspected PsA, and MRI of affected joints was ordered. Following evaluation and exclusion of pulmonary disease by chest radiography, disease-modifying therapy was initiated with methotrexate weekly and a short course of prednisone, along with folic acid supplementation. 

At the one-month follow-up, the patient reported significant improvement in joint pain and depressive symptoms following initiation of methotrexate and escitalopram. Her affect appeared more cheerful, and she expressed that her pain and energy levels had improved.

This timeline shows the series of evolving diagnoses and progression of disease leading up to the working diagnosis of PsA and the response to treatment (Figure 4).

**Figure 4:**
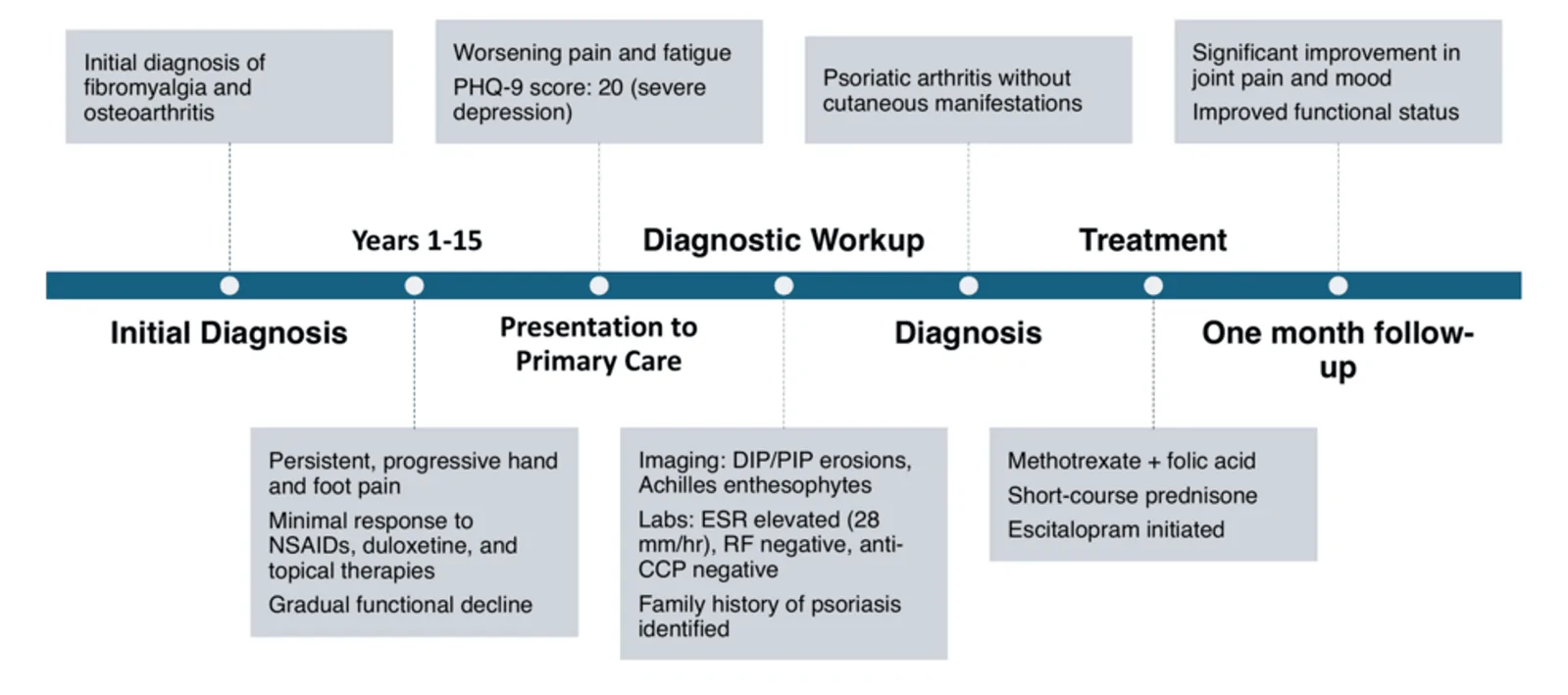
This timeline shows the series of evolving diagnoses and progression of disease leading up to the working diagnosis of psoriatic arthritis and the response to treatment. DIP: distal interphalangeal; PIP: proximal interphalangeal; ESR: erythrocyte sedimentation rate; RF: rheumatoid factor; anti-CCP: anti-cyclic citrullinated peptide; PHQ-9: Patient Health Questionnaire-9

Magnetic resonance imaging of affected joints had been obtained, but results were pending at the time of follow-up, representing a limitation. Continued rheumatology follow-up was planned, but the patient had not been seen at this time.

## Discussion

This case highlights long-standing musculoskeletal symptoms preceding eventual recognition of suspected PsA. A 15-year history of symptoms initially attributed to osteoarthritis and fibromyalgia demonstrates the importance of maintaining a broad differential diagnosis in patients with refractory pain. It should be noted that this timeline reflects the duration of symptoms rather than a confirmed 15-year course of untreated inflammatory disease, as earlier records demonstrating active inflammatory arthritis were not available.

Diagnostic delay 

Diagnostic delay in PsA results in postponing initiation of conventional synthetic disease-modifying antirheumatic drugs (csDMARDs), which is associated with inflammation, irreversible joint damage, functional impairment, and mental health decline [4]. This case illustrates a prolonged symptomatic period consistent with these documented consequences; however, the available data cannot confirm that inflammatory PsA was active and untreated throughout the full 15 years, or that the radiographic findings at presentation represent progression from a single continuous inflammatory process rather than a more recent onset superimposed on longstanding fibromyalgia and osteoarthritis.

PsA diagnosis remains difficult due to lack of specific biomarkers, variable presentation, and overlap with other diseases [4-6]. In noncutaneous presentations such as this case, diagnosis relies on recognizing clinical features such as joint involvement, enthesitis, imaging findings, and family history.

In this case, periarticular changes at the right ring finger and enthesopathic changes at the Achilles and plantar fascia insertions on imaging prompted consideration of an inflammatory etiology of symptoms.

Differential diagnosis

Misdiagnosis contributes significantly to delayed treatment in PsA [4]. In this case, fibromyalgia and osteoarthritis were initially diagnosed [4]. Differentiating between inflammatory and noninflammatory etiologies can be a starting point when evaluating chronic musculoskeletal pain, though several conditions merit consideration alongside PsA in a patient with this presentation.

Fibromyalgia is a noninflammatory condition characterized by widespread pain, lacks objective inflammatory or imaging findings, and is not associated with joint swelling, enthesitis, dactylitis, or psoriasis [7,10]. The presence of new bone formation and erosions on imaging in this patient indicated an inflammatory process not explained by fibromyalgia alone.

Distinguishing PsA from osteoarthritis is less straightforward, particularly given this patient's coexisting moderate degenerative changes; erosions are not exclusive to inflammatory arthritis, as erosive osteoarthritis can also produce marginal erosions, most classically in the DIP and PIP joints [10-12]. Features favoring PsA over erosive OA in this patient included the enthesopathic changes at the Achilles and plantar fascia insertions, which are not typical of osteoarthritis, along with the family history of psoriasis. Coexistence of osteoarthritis and PsA in the same patient is also possible and cannot be excluded here, given her age and the degenerative changes noted on imaging.

RA, including seronegative RA, was also considered. RA more typically involves the proximal joints (e.g., MCP, wrist), whereas PsA more typically involves the distal joints, though this distinction is not absolute and both patterns can overlap, as seen in this patient. Anti-CCP antibody positivity, rather than a negative result, supports a diagnosis of RA [4,10]; this patient's negative anti-CCP and negative rheumatoid factor argued against RA, and in combination with a family history of psoriasis and enthesopathic findings, supported PsA as the more likely diagnosis.

Peripheral spondyloarthritis without psoriasis and crystal arthropathy (e.g., gout, calcium pyrophosphate deposition disease) were also part of the differential given the distal joint symptoms and Achilles insertion tenderness. However, the strong family history of psoriasis and overall clinical and radiographic findings increased the suspicion of PsA over peripheral spondyloarthritis. Crystal arthropathy was considered less likely given the chronic, progressive symptom pattern without documented acute episodic attacks, podagra, or radiographic chondrocalcinosis. No synovial fluid analysis was performed; therefore, crystal arthropathy could not be definitively excluded. 

We note that the CASPAR juxta-articular new bone formation criterion was met based on the radiologist's impression of periarticular inflammatory change at the ring finger, rather than a finding described in explicit CASPAR terminology. We recommend that future application of CASPAR in similar cases include radiologic characterization using CASPAR-specific language where feasible, to reduce interpretive ambiguity in classification.

Psychological sequelae of delayed diagnosis

Chronic pain is strongly associated with depression through both biological and psychosocial mechanisms. Biological mechanisms include dysregulation of neurotransmitters such as serotonin, norepinephrine, substance P, and corticotropin-releasing factor. Patients with persistent pain are more likely to have maladaptive coping mechanisms that negatively influence pain and depression severity [13,14]. These negatively reinforced mechanisms include catastrophizing, feelings of helplessness, and low self-efficacy, which increase depressive and pain symptoms, further exacerbating this cycle [13,14].

In this case, PHQ-9 scores worsened from 14 to 20 over three years, indicating progression from moderate to severe depression, and improved at one-month follow-up. This improvement should be interpreted cautiously: methotrexate and a short course of prednisone were initiated at the same time that duloxetine was discontinued and escitalopram was started, so improvement in pain or mood cannot be confidently attributed to any single intervention. The temporal association is nonetheless consistent with the broader literature describing a bidirectional relationship between chronic inflammatory disease and mental health, and supports the importance of routine depression screening in patients with chronic pain.

Disease activity and treatment

At presentation, the patient reported chronic pain involving the hands and feet, with examination demonstrating tenderness of the DIP and PIP joints and bilateral Achilles tendon insertions. No peripheral edema was present. Plain radiography of the right hand demonstrated moderate degenerative changes of the digits, with cortical erosions and periarticular changes at the DIP and PIP joints of the right ring finger, read by radiology as consistent with an inflammatory arthropathy. Rheumatoid factor and anti-CCP antibodies were negative. Although an ESR had previously been obtained, standardized measures of PsA disease activity, including formal swollen and tender joint counts, a patient-reported pain score, CRP, validated enthesitis indices, functional assessment, and DAPSA, were not documented and therefore could not be retrospectively reported.

Baseline laboratory evaluation was obtained prior to initiation of methotrexate and was within acceptable limits for treatment. A chest radiograph was also obtained as part of the pre-immunosuppressive treatment evaluation. Treatment was initiated with oral methotrexate 12.5 mg once weekly (five 2.5-mg tablets) and a 12-day prednisone taper consisting of 30 mg daily for four days, 20 mg daily for four days, and 10 mg daily for four days. Folic acid supplementation was subsequently initiated while methotrexate therapy was continued. The patient was counseled regarding medication-related adverse effects and continued monitoring.

At the approximately one-month follow-up, the patient reported improvement in joint pain and mood while continuing methotrexate and awaiting MRI results. However, this improvement cannot be attributed to methotrexate alone because prednisone was administered concurrently and antidepressant therapy was also modified during this period. Additionally, quantitative disease-activity measures were not available at follow-up to objectively characterize the magnitude of improvement.

Clinical implications

Although the delays in diagnosis and treatment have been presented in the literature for over a decade, improvements remain unimpressive [15]. This case underscores the value of maintaining a high index of suspicion for inflammatory arthropathies in patients with persistent, treatment-refractory musculoskeletal symptoms, and of pursuing a comprehensive history, examination, and appropriate imaging even when classic cutaneous features are absent, rather than establishing a proven causal chain between this patient's specific diagnostic delay, her structural progression, and her psychological decline. Early recognition, interdisciplinary collaboration, and routine mental health screening remain essential to reducing diagnostic delay and facilitating timely initiation of therapy.

Osteopathic perspective 

From an osteopathic perspective, this case highlights the relationship between structure and function, with chronic inflammation contributing to both physical manifestations and psychological burden. This reinforces the bidirectional relationship between inflammatory disease and depression. Osteopathic manipulative treatment (OMT) may serve as an adjunct to restore physiologic balance and improve pain and overall well-being. Although more research is needed to establish its role in PsA, emerging evidence suggests effects on inflammatory and autonomic pathways that may help provide symptomatic relief and improve overall psychologic state [16].

## Conclusions

This case illustrates the diagnostic challenge posed by PsA in patients without a classic cutaneous presentation, and the prolonged symptomatic course that can accompany unrecognized inflammatory disease. While this case is consistent with the broader literature linking diagnostic delay to structural progression and psychological burden, it does not establish that PsA was present throughout the full symptomatic period or that the observed structural and psychological findings represent proven consequences of delayed diagnosis in this patient. Maintaining a broad differential and considering inflammatory arthropathy in patients with persistent, treatment-resistant musculoskeletal pain, even in the absence of psoriasis, may support earlier recognition. A comprehensive, patient-centered approach incorporating history, examination, and appropriate imaging may improve both physical and psychological outcomes in similar cases.
